# SF3B4 promotes ovarian cancer progression by regulating alternative splicing of RAD52

**DOI:** 10.1038/s41419-022-04630-1

**Published:** 2022-02-24

**Authors:** Yuchao Diao, Yingwei Li, Zixiang Wang, Shourong Wang, Peng Li, Beihua Kong

**Affiliations:** 1 Department of Obstetrics and Gynecology, Qilu Hospital, Cheeloo College of Medicine, Shandong University, Jinan, Shandong 250012 China; 2 Department of Obstetrics and Gynecology, the Affiliated Hospital of Qingdao University, Qingdao, 266000 Shangdong China; 3 School of Medicine, Cheeloo College of Medicine, Shandong University, Jinan, Shandong 250012 China

**Keywords:** Ovarian cancer, Prognostic markers

## Abstract

Many studies have proven that splicing factors are crucial for human malignant tumor development. However, as a classical splicing factor, the expression of SF3B4 is not clear, and its biological function needs to be further clarified in ovarian cancer (OC). We determined that SF3B4 was obviously upregulated and its high expression was associated with poor prognosis in OC patients. In vitro and in vivo assays suggested that SF3B4 overexpression promoted OC cell proliferation and mobility, and downregulation of SF3B4 had the opposite effect. Further studies found that miR-509–3p decreased SF3B4 mRNA expression by binding to the 3’ -UTR of SF3B4 directly. Importantly, we revealed that RAD52 was a potential target of SF3B4 through alternative splicing events analysis. Loss of SF3B4 led to decreased expression of RAD52, owing to intron 8 retention and generation of premature termination codons. Moreover, decreased expression of RAD52 partially counteracted the tumor-promoting effect of SF3B4 overexpression. In conclusion, our results suggested that SF3B4, negatively regulated by miR-509–3p, promoted OC progression through effective splicing of RAD52. Therefore, SF3B4 may be a promising biomarker and effective therapeutic target for OC.

## Introduction

OC is the most lethal gynecologic malignancy [1]. More than 60% of patients are diagnosed at an advanced stage due to a lack of early diagnostic technology and rapid progression [2], and the five-year survival rate is approximately 45% [2]. Although the advent of antiangiogenic drugs and PARP inhibitors can benefit some patients [3–5], they cannot fundamentally change the outcome of all OC patients. Therefore, the mechanism of initiation and development of OC needs to be elucidated further.

Alternative splicing (AS) can produce RNA isoforms and generate protein diversity [6]. In humans, more than 95% of genes exhibit AS [7]. Many diseases, including human malignant tumors, are associated with abnormal gene splicing [8]. Multiple studies have confirmed that some RBPs are involved in OC development. For example, RBP USP39 promotes OC progression by targeting HMGA2 [9]. IGF2BP3 and Lin28B are associated with drug resistance in OC patients [10]. SFPQ regulates the platinum response by modulating SRSF2 activity in OC [11]. SORBS2 suppresses immune evasion of OC [12].

SF3B is a component of the U2 small nuclear ribonucleoprotein (snRNP), which is involved in the splicing of pre-mRNA [13]. Studies have shown that SF3B1, SF3B2, and SF3B3 can regulate target gene expression by alternative splicing to promote cancer progression [14–16]. SF3B1 promotes the progression of endometrial cancer by regulating KSR2 RNA maturation [14]. SF3B2 drives prostate cancer progression by mediating target gene splicing [15]. In renal cancer, SF3B3 contributes to tumorigenic potential by regulating alternative splicing of EZH2 [16]. Importantly, the SF3B1 inhibitor FR901464 plays an anticancer role in a variety of cancers [14, 17–19]. SF3B4 is an important subunit of SF3B [13], and it is involved in pre-mRNA splicing and regulates cell signaling, transcription and translation [20]. SF3B4 promotes hepatocellular carcinoma progression by regulating KLF4 [21]. SF3B4 serves as an oncogene in esophageal cancer [22]. In contrast, SF3B4 acts as a cancer suppressor in pancreatic cancer [23]. The expression and function of SF3B4 are inconsistent in different tumors. SF3B4 was significantly increased and related to worse prognosis of OC patients through bioinformatics analysis. However, the potential mechanism of SF3B4 in the development of OC remains to be explored.

In this study, we uncovered that SF3B4 protein is upregulated and intimately related to worse prognosis in OC patients. Furthermore, we showed that SF3B4 facilitated the malignant potential of OC cells both in vitro and in vivo. In addition, we revealed that SF3B4 was regulated by miRNA-509–3p and maintained efficient splicing of RAD52. Thus, SF3B4 may be a prognostic biomarker and therapeutic target of OC patients.

## Results

### SF3B4 is overexpressed in OC and correlates with poor prognosis

**Fig. 1:**
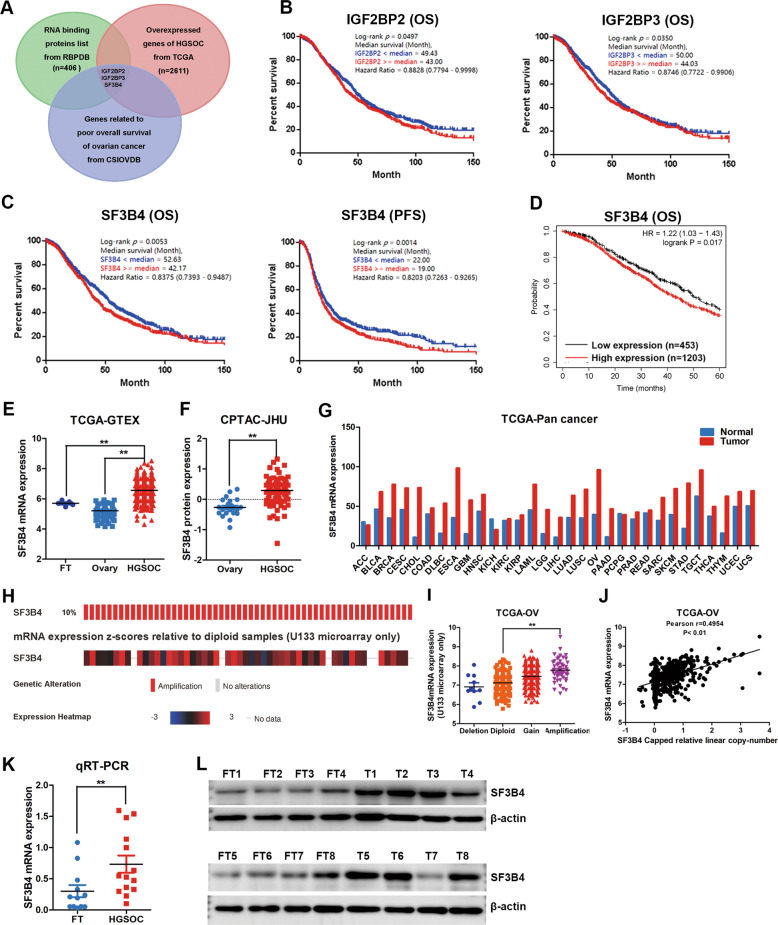
SF3B4 is upregulated in ovarian cancer and correlates with poor prognosis. **A** Venn diagram showing the classical RNA binding proteins from RBPDB, which are overexpressed in TCGA and associated with poor prognosis from CSIOVDB of ovarian cancer. **B** Kaplan–Meier analysis of the effect of IGF2BP2 and IGF2BP3 expression on the overall survival of ovarian cancer patients from CSIOVDB (*n* = 1868). **C**, **D** Kaplan–Meier analysis showed the effect of SF3B4 expression on the overall survival and progression-free survival of ovarian cancer patients from CSIOVDB and K-M plotter. The samples of overall survival and progression-free survival analysis from CSIOVDB were 1868 and 1516. The samples of overall survival analysis from K-M plotter were 1656. **E** Relative mRNA expression of SF3B4 in ovarian cancer (*n* = 426), normal ovary (*n* = 88) and fallopian tube (*n* = 5) tissues from TCGA-GTEX. **F** Relative protein expression of SF3B4 in ovarian cancer (*n* = 85) and normal ovary (*n* = 22) tissues from CPTAC-JHU. **G** Analysis of differential expression of SF3B4 across TCGA pan-cancers. **H** Genetic alterations of SF3B4 in ovarian cancer in the cohort from TCGA Firehose Legacy (*n* = 579). **I** Relative SF3B4 mRNA expression of samples with different copy number variation statuses from TCGA (Amplification: *n* = 50; Gain: *n* = 261; Diploid: *n* = 191; Deletion: *n* = 10). **J** Correlation analysis between SF3B4 amplification and mRNA expression in TCGA ovarian cancer samples (*n* = 512). **K** qRT-PCR analysis of SF3B4 mRNA expression between ovarian cancer (*n* = 14) and fallopian tube (*n* = 12) tissues. **L** Western blotting showed the differences in the SF3B4 protein levels between ovarian cancer (*n* = 8) and fallopian tube (*n* = 8) tissues. *P* value was obtained by Log-rank test (**B**, **C**, **D**) or Unpaired *t*-test (**E**, **F**, **I**, and **K**). *P < 0.05, **P < 0.01.

To search for critical splicing factors involved in the initiation and development of OC, overlapping analysis of 2611 upregulated genes, genes related to poor prognosis, and 406 classical RNA-binding proteins revealed 3 potential genes, including IGF2BP2, IGF2BP3, and SF3B4 (Fig. 1A). Kaplan–Meier analysis revealed that overexpression of IGF2BP2 and IGF2BP3 indicated poor overall survival (OS) in OC patients (Fig. 1B). SF3B4 was found to be associated not only with poor OS but also with worse progression-free survival (PFS) from CSIOVDB (Fig. 1C). The same result showed that higher expression of SF3B4 correlated with poor OS from Kaplan–Meier plotter (Fig. 1D).

To determine the expression of SF3B4 in OC, we used TCGA and GTEX databases to examine SF3B4 expression and found that it was obviously upregulated in most OC samples (Fig. 1E). The same result for SF3B4 protein expression was obtained from the CPTAC database (Fig. 1F). Importantly, SF3B4 was overexpressed in various cancers by pan-cancer analysis (Fig. 1G), indicating that SF3B4 is involved in the development and progression of human cancers. Moreover, approximately 10% of OC cases had SF3B4 amplification (Fig. 1H), and its mRNA expression was positively correlated with its amplification (Fig. 1I, J). Next, we validated SF3B4 mRNA and protein expression by qRT–PCR and WB in fresh-frozen tissues, and SF3B4 was frequently upregulated in OC samples compared with FT tissues (Fig. 1K, L). These data indicate that SF3B4 is upregulated in OC and is a potential prognostic biomarker for OC patients.

### SF3B4 promotes the proliferation and mobility of OC cells in vitro

**Fig. 2:**
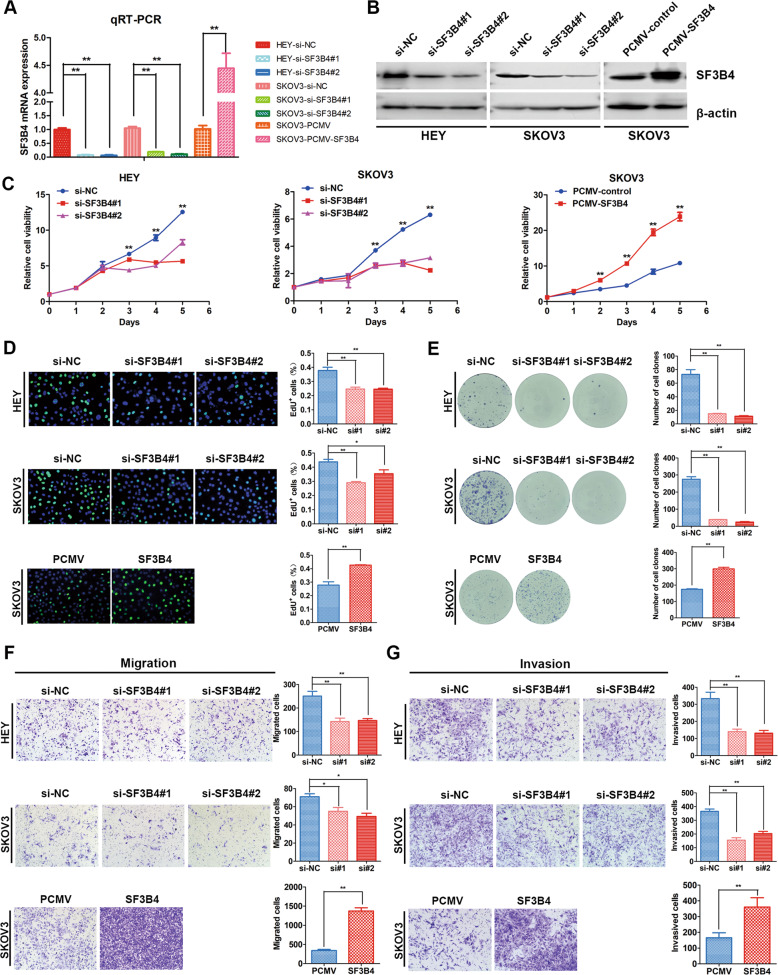
SF3B4 promotes proliferation, migration and invasion of ovarian cancer cells. **A**, **B** SF3B4 knockdown and overexpression efficiency was confirmed by qRT-PCR (*n* = 3 biologically independent samples) and western blotting. **C** Growth curve showed the growth of ovarian cancer cells upon SF3B4 knockdown and overexpression (*n* = 3 biologically independent samples). **D** EdU assay showed the proliferation ability of ovarian cancer cells after SF3B4 knockdown and overexpression (*n* = 3 biologically independent samples). **E** The effect of SF3B4 on colony formation in ovarian cancer cells (*n* = 3 biologically independent samples). **F**, **G** In HEY and SKOV3 cells, up and downregulation of SF3B4 affected migration and invasion capacity (*n* = 3 biologically independent samples). *P*-value was obtained by Unpaired *t*-test. Results represent the mean ± SD of three independent experiments. *P < 0.05, **P < 0.01.

To investigate the effect of SF3B4 in OC cells, we transiently transfected HEY, A2780 and SKOV3 cells with SF3B4 siRNA to knock down SF3B4. Then, we established stable SF3B4-overexpressing SKOV3 cell lines. qRT–PCR and WB showed that the mRNA and protein expression decreased significantly after SF3B4 knockdown. In contrast, overexpression of SF3B4 obviously upregulated SF3B4 mRNA and protein levels (Figs. 2A, B and S1A, B).

Then, we performed MTT and EdU assays and found that overexpression of SF3B4 obviously enhanced the proliferation of OC cells, whereas SF3B4 inhibition slowed proliferation (Figs. 2C, D and S1C, D). In addition, a clonogenic assay was performed, and we found that SF3B4 dramatically promoted the clonogenic capacity of SKOV3 cells. In contrast, SF3B4 knockdown led to reduced clonogenicity (Fig. 2E). Furthermore, we measured migration and invasion ability with a Transwell assay, and our results showed that upregulation of SF3B4 increased the migration and invasion capacity in OC cells, whereas downregulation of SF3B4 had the opposite effect (Figs. 2F, G and S1E). These results suggest that SF3B4 enhances the proliferation ability and mobility of OC cells in vitro.

### SF3B4 depletion suppresses tumorigenic ability in vivo

**Fig. 3:**
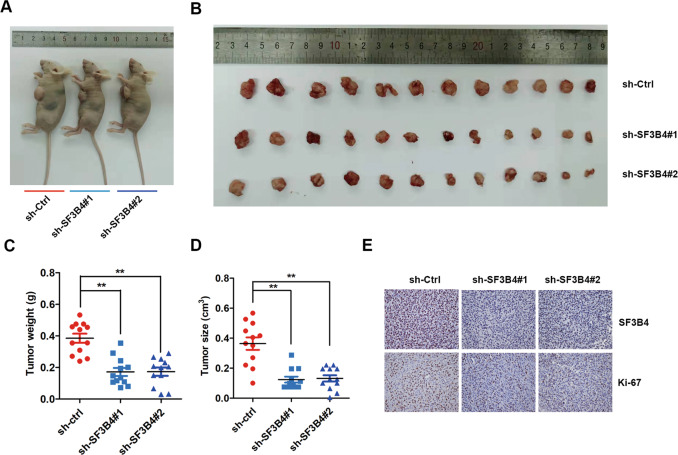
Knockdown of SF3B4 suppresses xenograft tumor growth in vivo. **A**, **B** Images of tumors isolated from subcutaneous implantation of six nude mice bilaterally with SF3B4 knockdown and control HEY cells (*n* = 12 per group). **C** Tumor weight statistics. **D** Tumor size statistics. **E** Immunohistochemical staining images of SF3B4 and Ki-67 in xenograft tumors from SF3B4 knockdown and control cells treated nude mice. *P*-value was obtained by Unpaired *t*-test. Results represent the mean ± SD. *P < 0.05, **P < 0.01.

To clarify the biological function of SF3B4 in the tumorigenicity of OC cells in vivo, we constructed SF3B4 stable downregulated cell lines (sh-SF3B4#1 and 2) and control vector cell lines (sh-Ctrl). Then, sh-SF3B4 and sh-Ctrl OC cells were injected into nude mice subcutaneously. All mice were sacrificed, and subcutaneous tumors were dissected 3 weeks later (Fig. 3A, B). Tumor size was obviously smaller, and tumor weight was also much lighter in the sh-SF3B4 group than in the sh-Ctrl group (Fig. 3C, D). Then, we examined the expression of SF3B4 and the proliferation protein Ki-67 in our established xenograft model. The immunohistochemical staining results showed that SF3B4 expression was obviously downregulated in the sh-SF3B4 group (Fig. 3E). Similarly, the level of Ki-67 was significantly decreased in the sh-SF3B4 group compared with the control group (Fig. 3E). These findings demonstrate that SF3B4 promotes OC growth in vivo.

### RAD52 is a critical downstream target of SF3B4 based on RNA-seq analysis

**Fig. 4:**
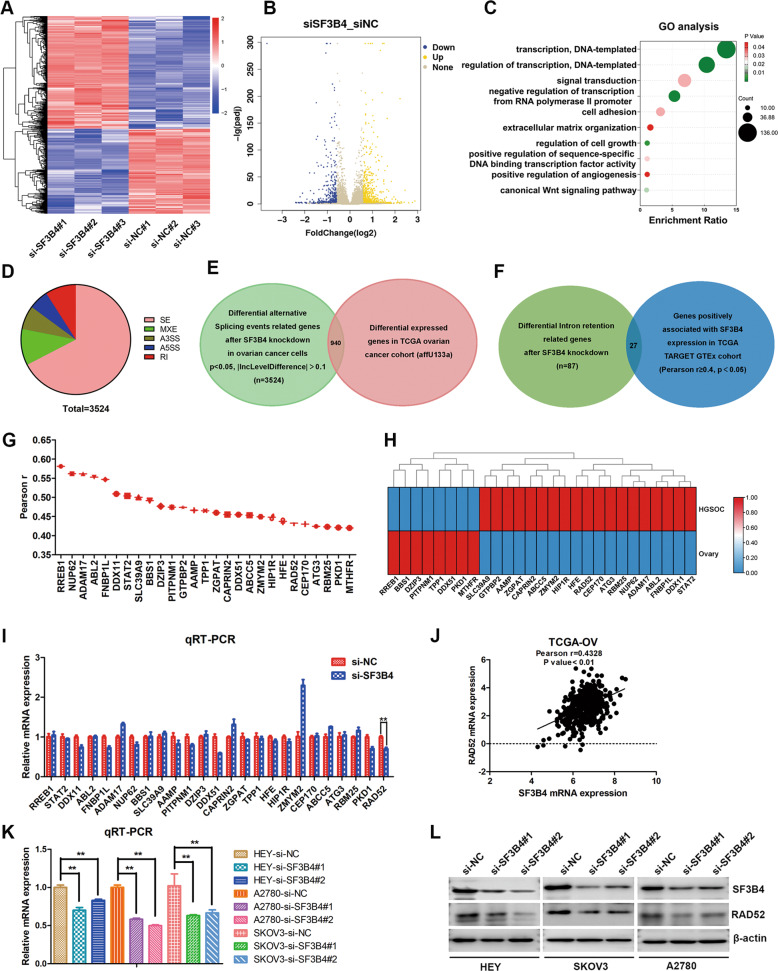
RAD52 is a downstream target of SF3B4. **A**, **B** Heatmap and volcano plot of differential expression genes from RNA-seq analysis of SF3B4 knockdown and control in HEY cells. **C** Bubble diagram showing the results of GO analysis of differential expression genes described in (**A**). **D** Pie chart of differential alternative splicing events in HEY cells response to SF3B4 knockdown. **E** Venn diagram showing 3524 genes related to differential alternative splicing events after SF3B4 knockdown, of which 940 genes are differential expressed in TCGA ovarian cancer. **F** Venn diagram showing 27 genes positively correlated with SF3B4 expression in TCGA TARGET GTEx cohort and involved in intron retention events from RNA-seq analysis. **G** Schematic diagram showing correlation analysis between SF3B4 and genes described in (**F**). **H** Heatmap of selected genes described in (**F**). **I** qRT-PCR analysis of mRNA expression of selected genes described in (**F**) after SF3B4 knockdown in HEY cells. **J** Correlation analysis between SF3B4 and RAD52 mRNA expression in TCGA ovarian cancer (*n* = 426). **K** qRT-PCR analysis of RAD52 mRNA expression after SF3B4 knockdown in ovarian cancer cells (*n* = 3 biologically independent samples). **L** Western blotting showed the SF3B4 and RAD52 protein levels after SF3B4 knockdown. *P*-value was obtained by Unpaired *t*-test. *P < 0.05, **P < 0.01.

To investigate the mechanism by which SF3B4 promotes the malignancy of OC. RNA-seq was applied to analyze the critical downstream targets after SF3B4 knockdown in OC cells. The differentially expressed genes (DEGs) heatmaps and volcano plots are represented in Fig. 4A, B ( | log2 FC | ≥ 1 and *p* < 0.05). There were 665 upregulated and 501 downregulated genes. We used Gene oncology analysis (https://david.ncifcrf.gov/) to evaluate the biology functions of DEGs, and we found that the biological processes were enriched in regulation of transcription, cell adhesion, extracellular matrix organization and regulation of cell growth (Fig. 4C).

Because SF3B4 is a splicing factor, we used rMATS software to perform alternative splicing events analysis. Then, we found 3524 genes related to differential alternative splicing events after SF3B4 knockdown, including 2376 skipped exons and 320 retained introns (Fig. 4D). 940 genes were identified to be differential expressed in TCGA OC and involved in the differential alternative splicing events (Fig. 4E). Studies have shown that SF3B family could regulate the expression of downstream genes by inclusion of intron [14, 15]. It was still unclear whether SF3B4 regulated expressions of downstream target genes through intron retention in OC. So, we identified 27 critical genes both involved in intron retention events (*p* < 0.05, |delInclevel | > 0.1) after SF3B4 knockdown and positively correlated with SF3B4 expression (Pearson *r* ≥ 0.4, *p* < 0.05) in TCGA TARGET GTEx cohort (Fig. 4F). Correlation and differential expressed analysis between SF3B4 and 27 selected genes in TCGA OC were demonstrated in Fig. 4G, H. Next, we verified the expression of these 27 selected genes after SF3B4 knockdown in HEY cells through qRT–PCR, and the results showed that 6 genes (DDX11, FNBP1L, PITPNM1, DDX51, PKD1 and RAD52) were obviously downregulated (Fig. 4I). RAD52 is involved in DNA damage repair, and plays an oncogene role in a variety of tumors. More importantly, the inhibitor of RAD52 has been developed, and it improves therapeutic effect of BRCA-deficient malignancies [24]. Correlation analysis between SF3B4 and RAD52 was shown in Fig. 4J. Therefore, we selected RAD52 for further research. Then, we verified the effect of SF3B4 knockdown on RAD52 in different OC cells, and the expression of RAD52 mRNA and protein was obviously decreased (Fig. 4K, L). In summary, SF3B4 regulates RAD52 expression in OC cells, and RAD52 is a critical downstream target of SF3B4.

### SF3B4 facilitates efficient splicing of RAD52 intron 8

**Fig. 5:**
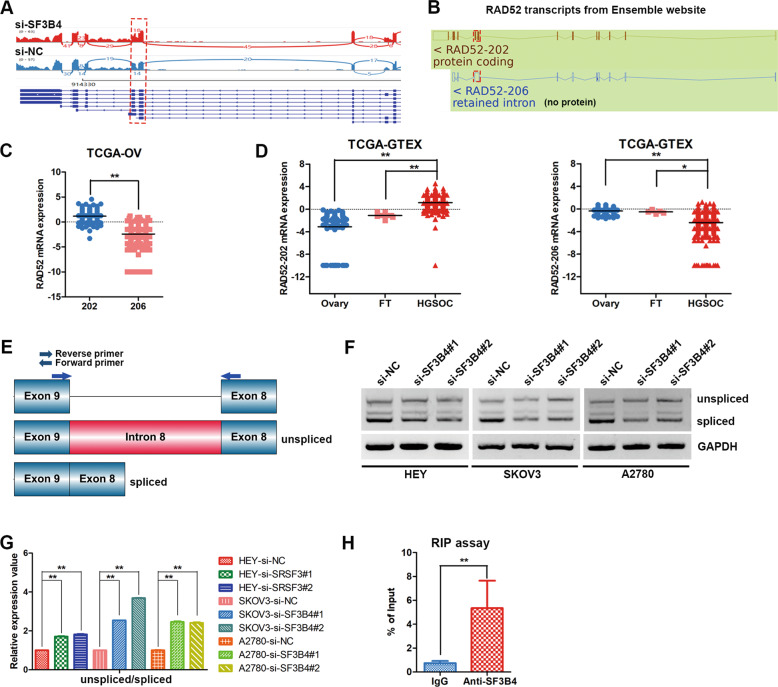
SF3B4 facilitates efficient splicing of RAD52. **A** Sashimi plot visualization of RNA-seq reads mapping to RAD52 in HEY cells in response to SF3B4 knockdown. **B** Schematic diagram showing two splicing variants of the RAD52 mRNA transcript identified from the Ensemble genome browser. **C**, **D** Relative mRNA expression of RAD52 splicing variants described in (**B**) from TCGA ovarian cancer database (202: *n* = 426; 206: *n* = 426; HGSOC: *n* = 426; Ovary: *n* = 88; FT: *n* = 5). **E** Schematic diagram showing the position of intron retention and primers that used for RT-PCR (*n* = 3 biologically independent samples). F, **G** Relative mRNA expression of RAD52 transcripts in ovarian cancer cells after SF3B4 knockdown (*n* = 3 biologically independent samples). **H** The interaction between SF3B4 and RAD52 mRNA was validated by RIP-PCR in HEY cells (*n* = 3 biologically independent samples). *P*-value was obtained by Unpaired *t*-test. *P < 0.05, **P < 0.01.

To explore the splicing changes of RAD52 after SF3B4 knockdown. A Sashimi plot was used to visualize the RNA-seq reads of RAD52, and intron 8 retention of RAD52 was found to be obviously increased after SF3B4 knockdown in HEY cells (Fig. 5A). Ensemble genome browser was applied to analyze the splice variants of RAD52 mRNA. We found an intron (intron 8) retained in the RAD52–206 transcript (unspliced intron 8) by comparing the noncoding transcript variant RAD52–206 and the protein-coding transcript variant RAD52–202 (spliced intron 8) (Fig. 5B). Retained intron 8 of RAD52 can introduce a stop codon (TAA) in an open reading frame, resulting in premature termination of translation. RAD52–202 and RAD52–206 transcripts were both present in the TCGA OC cohort, and RAD52–206 transcript expression was lower than RAD52–202 in OC (Fig. 5C). Then, we examine the expression of RAD52–202 and RAD52–206 transcripts between OC and normal control tissues in the TCGA and GTEX databases, and the result showed that RAD52–202 transcript was obviously upregulated and RAD52–206 transcript was obviously downregulated in most OC samples comparing normal ovary and fallopian tube tissues (Fig. 5D).

Next, RT–PCR was performed to using primers that span the intron 8 within exons 8 and 9, and we found that knockdown of SF3B4 significantly increased the unspliced transcript variant and reduced the spliced transcript variant (Fig. 5E–G). The RIP–PCR results indicated that RAD52 expression was significantly higher in SF3B4 pulldowns than in IgG controls (Fig. 5H), suggesting that the SF3B4 protein bound to RAD52 mRNA specifically. In conclusion, SF3B4 regulates RAD52 expression through regulating intron retention.

### Knockdown of RAD52 impaired the phenotype of SF3B4 overexpression

**Fig. 6:**
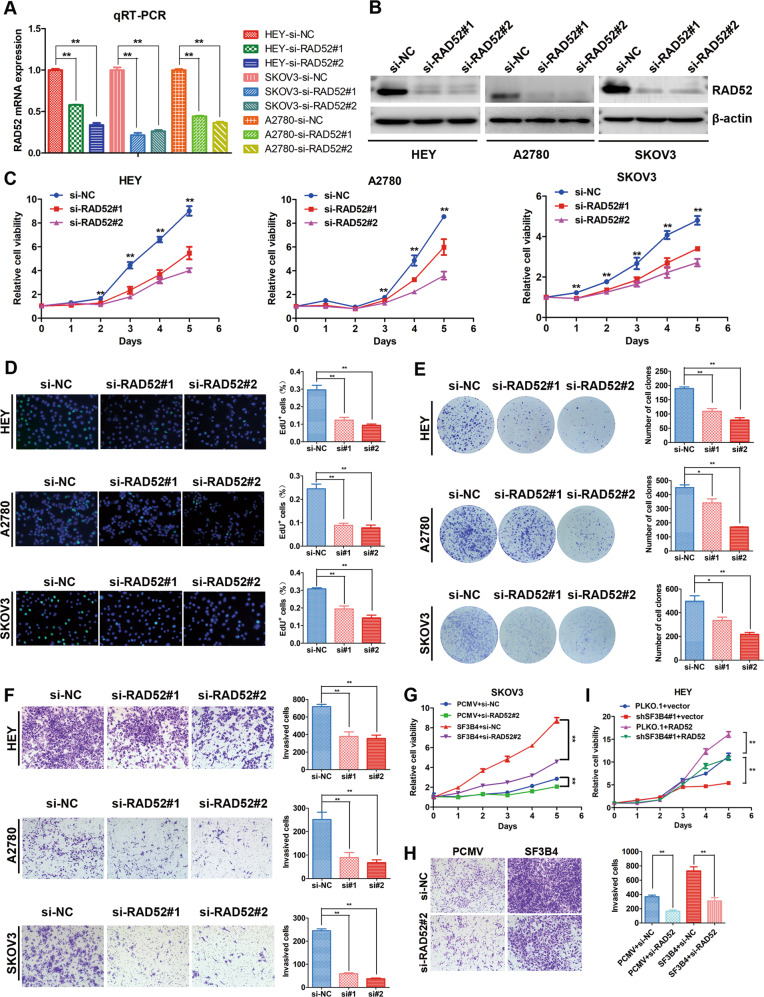
RAD52 promotes cell proliferation and mobility in ovarian cancer cells. **A**, **B** RAD52 knockdown efficiency was confirmed by qRT-PCR (*n* = 3 biologically independent samples) and western blotting. **C** Growth curve and (**D**) EdU assay and (**E**) clonogenic assay were used to evaluate the effect of SF3B4 on the proliferation of HEY, A2780, and SKOV3 cells (*n* = 3 biologically independent samples). **F** Transwell assay showed the invasion capacity of ovarian cancer cells upon RAD52 knockdown (*n* = 3 biologically independent samples). **G**, **H** Different growth and invasion abilities associated with different expression levels of SF3B4 and RAD52 in SKOV3 cells (*n* = 3 biologically independent samples). **I** Growth curve assay was used to investigate the potential of RAD52 to rescue the loss of SF3B4 in ovarian cancer cells (*n* = 3 biologically independent samples). *P*-value was obtained by Unpaired *t*-test. *P < 0.05, **P < 0.01.

Next, we investigated the biological function of RAD52 by transiently transfecting OC cells with siNC and RAD52-targeted siRNA to knock down RAD52. The mRNA and protein expression levels of RAD52 were significantly decreased (Fig. 6A, B). The EdU and MTT assays showed that downregulation of RAD52 obviously weakened the proliferation capacity of OC cells (Fig. 6C, D). Then, we performed a colony formation assay, and the results revealed that RAD52 inhibited the colony formation potential of OC cells (Fig. 6E). In addition, the Transwell assay indicated that a decrease in RAD52 weakened the migration and invasion ability of OC cells (Figs. 6F and S2A).

To explore whether RAD52 is involved in SF3B4-mediated malignancy of OC cells, we transiently transfected control or RAD52-targeted siRNA into SF3B4-overexpressing SKOV3 cells. Then, we found that knockdown of RAD52 in SF3B4-overexpressing SKOV3 cells successfully impaired the enhanced capacity of cell proliferation, migration and invasion (Figs. 6G, H and S2B). Overexpression of RAD52 rescued the inhibition of proliferation induced by silencing SF3B4 (Fig. 6I). In summary, RAD52 is a major target of SF3B4 and mediates the biological function of SF3B4 in OC.

### SF3B4 expression is negatively regulated by miR-509–3p

**Fig. 7:**
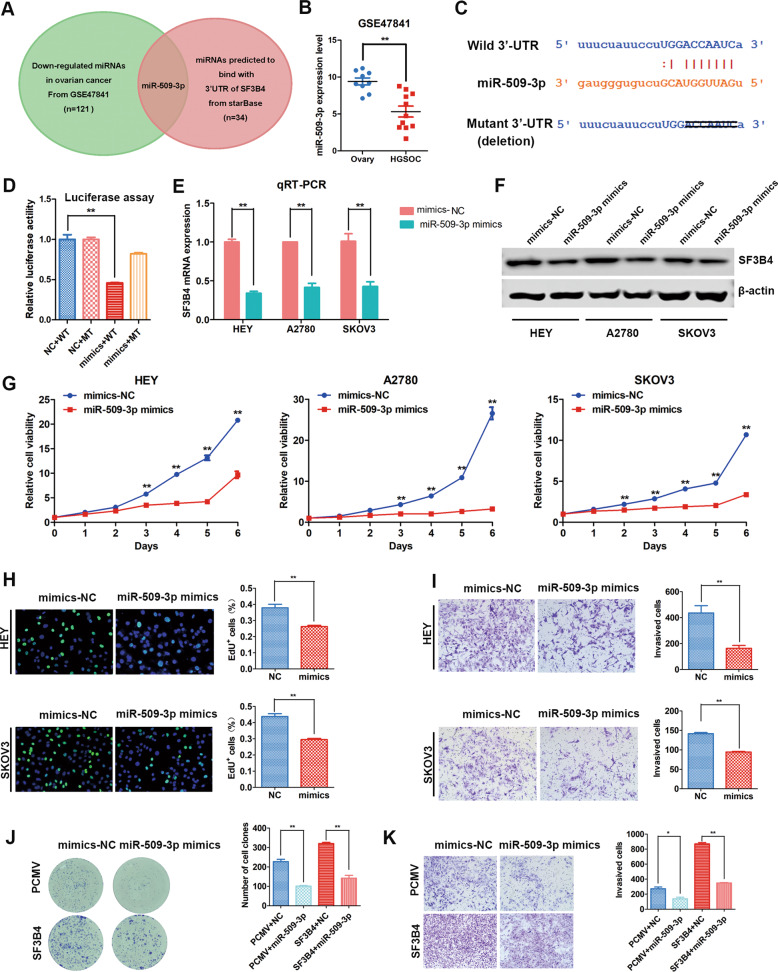
miR-509–3p directly targets SF3B4 and suppress proliferation and invasion of ovarian cancer cells. **A** Venn diagram showing the microRNA predicted to bind with 3’UTR of SF3B4 from starBase (*n* = 34) and downregulated in ovarian cancer from GSE47841(*n* = 121). **B** Relative miR-509–3p expression in ovarian cancer (*n* = 11) and normal ovary (*n* = 9) tissues from GSE47841. **C** Potential binding sequence of miR-509–3p in the 3’UTR of SF3B4. **D** Luciferase reporter assays for SF3B4 and miR-509–3p (mimics, miR-509-3p mimics; WT, SF3B4 3’UTR wild sequence; MT, SF3B4 3’UTR mutant sequence) (*n* = 3 biologically independent samples). **E** qRT-PCR analysis of SF3B4 mRNA expression after miR-509–3p overexpression in ovarian cancer cells (*n* = 3 biologically independent samples). F Western blot analysis of SF3B4 protein expression after miR-509–3p overexpression in ovarian cancer cells. **G** Growth curve showed the effect of miR-509–3p overexpression on the growth ability of HEY, A2780, and SKOV3 cells (*n* = 3 biologically independent samples). **H** EdU assay showed the proliferation ability of ovarian cancer cells upon miR-509–3p overexpression (*n* = 3 biologically independent samples). **I** Change of invasion potential of HEY and SKOV3 cells after upregulation of miR-509–3p (*n* = 3 biologically independent samples). **J**, **K** Different colony formation and invasion abilities associated with different expression levels of miR-509–3p and SF3B4 in SKOV3 cells. (*n* = 3 biologically independent samples). *P* value was obtained by Unpaired *t*-test. *P < 0.05, **P < 0.01.

To explore the upstream regulatory mechanism of SF3B4, we found 34 miRNAs that might bind to the 3’UTR of SF3B4 through StarBase (http://starbase.sysu.edu.cn/). Then, we found that 121 miRNAs were downregulated in OC tissues from GSE47841. Overlapping analysis found that only miR-509–3p, whose expression was decreased in OC, could bind to the 3’UTR of SF3B4 (Fig. 7A, B). The wild type and mutant potential binding site of miR-509–3p in the 3’UTR of SF3B4 was shown in Figs. 7C and S3A. Then, we used a dual-luciferase reporter gene assay to confirm whether miR-509–3p could directly modulate SF3B4 expression. We constructed wild-type and mutant reporter plasmids containing the SF3B4–3’UTR. The results showed that miR-509–3p overexpression significantly decreased the luciferase activity in wild type 3’UTR, whereas the luciferase activity in mutant 3’UTR was enhanced after miR-509–3p overexpression compared with wild type (Fig. 7D). These results implied that miR-509–3p directly bound to the 3’UTR of SF3B4.

Moreover, to further prove whether SF3B4 expression is regulated by miR-509–3p, we transiently transfected miR-509–3p mimics into OC cells, and the expression of SF3B4 mRNA and protein was obviously decreased (Fig. 7E, F). Above all, these results suggested that miR-509–3p serves as a negative regulator of SF3B4 and decreases its expression in OC cells.

### miR-509–3p inhibits the progression of OC cells by targeting SF3B4

We transiently transfected miR-509–3p mimics to investigate the biological function of miR-509–3p. The results of the MTT and EdU assays showed that miR-509–3p obviously decreased the proliferation ability of OC cells (Figs. 7G, H Fig. S3B). The clone formation capacity was reduced dramatically after transfection with miR-509–3p mimics (Fig. S3C). Next, Transwell assays were performed, and the results showed that miR-509–3p mimics obviously weakened the mobility of OC cells (Figs. 7I and S3D, E).

We further confirmed whether miR-509–3p weakened the malignant behavior of OC cells through SF3B4. We transiently transfected miR-509–3p mimics/vector, miR-509–3p mimics/pCMV-SF3B4, NC/vector or NC/pCMV-SF3B4 into SKOV3 cells at the same time. Then, we found that upregulation of SF3B4 reversed the inhibitory effect of miR-509–3p in SKOV3 cells (Figs. 7J, K and S3F, G). In summary, these results reveal that miR-509–3p inhibits the progression of OC cells by regulating SF3B4.

## Discussion

In recent years, increasing evidence has shown that RBPs play important roles in the initiation and development of multiple tumors [25, 26]. SF3B4 is a subunit of the classical splicing factor family SF3B [13], and its abnormal expression is involved in tumorigenesis and progression. SF3B4 promotes cell proliferation and metastasis, and acts as an early-stage diagnostic marker in hepatocellular cancer [21, 27], and SF3B4 could facilitate lymphatic progression of esophageal cancer [22]. However, SF3B4 inhibits growth and migration abilities of pancreatic cancer cells [23]. In this study, we found that SF3B4 was abnormally overexpressed and its high expression was associated with poor OS and PFS in OC. The results suggest that SF3B4 could be a vital prognostic biomarker of OC.

Next, we studied the regulatory effect of SF3B4 on the biological function of OC cells and found that SF3B4 knockdown reduced the proliferation and mobility. In contrast, SF3B4 overexpression promoted aggressiveness. Meanwhile, we established a mouse xenograft tumor model to prove that the growth of subcutaneous tumors was much slower when SF3B4 was knocked down. These results are consistent with a study in hepatocellular cancer [21]. Our study indicates for the first time that SF3B4 acts as an oncogene in OC.

Effective splicing of pre-mRNA is essential for the production of mature and functional mRNA [28]. However, the splicing function of SF3B4 has not been clarified, and its targets in OC need further identification. Therefore, we performed RNA-seq to search for alternative splicing events. rMAT analysis and RT-PCR validation found that intron 8 of RAD52 was dramatically retained after SF3B4 knockdown in OC cells.

RAD52 is involved in DNA damage repair, including single-strand annealing and homologous recombination repair of double-strand breaks [29]. RAD52 knockdown obviously weakens cell proliferation in lung squamous cell carcinoma [30]. RAD52 promotes cell proliferation capacity and mobility in HCC [31]. Elevated RAD52 expression is related to worse DFS in patients with rectal cancer [32]. In this paper, we confirmed that the progression of OC cells was reduced dramatically after RAD52 knockdown. RAD52 knockdown impaired the phenotypic change caused by SF3B4 overexpression.

miRNAs are small noncoding RNAs and regulate approximately 30–60% of human protein-coding genes [33]. Studies have shown that miR-509–3p is associated with platinum drug sensitivity [34] and inhibits metastasis in OC [35]. In this study, we found for the first time that miR-509–3p was confirmed to suppress SF3B4 expression by binding to its 3’UTR.

In conclusion, we confirmed the expression, biological function and regulatory mechanism of SF3B4 in OC for the first time. In this study, we found that SF3B4 was upregulated and related to poor OS and PFS in OC patients. SF3B4 promoted OC cell proliferation, migration and invasion by regulating alternative splicing of RAD52. Moreover, SF3B4 was a direct target gene of miR-509–3p in OC. Taken together, the miR-509–3p/SF3B4/RAD52 axis regulates the malignant biological behavior of OC cells.

## Materials and methods

### Patients and tumor samples

OC and fallopian tube (FT) samples were obtained from surgical resections at the Department of Gynecology, Qilu Hospital, Shandong University. FT tissues were extracted from patients with benign diseases who underwent total hysterectomy and bilateral salpingectomy. OC specimens were extracted from patients with primary OC and without previous chemotherapy treatment or surgery. We performed qRT–PCR and western blotting analysis using 22 fresh-frozen OC and 20 FT tissues. All patients in this study provided informed consent. Ethical approval was obtained from the Ethics Committee of School of Medicine, Shandong University (SDULCLL2019–1-09).

### Clinical prognosis analysis from public database

The effect of SF3B4 mRNA expression on the clinical prognosis (Overall survival and Progression-free survival) was analyzed using the data from CSIOVDB. The ovarian cancer histologies from CSIOVDB (http://csiovdb.mc.ntu.edu.tw/CSIOVDB.html) include clear cell, endometrioid, mucinous, low-grade serous and serous. The relationship between SF3B4 mRNA expression and overall survival was evaluated by the online Kaplan-Meier plotter. Ovarian cancer data on this website does not limit ovarian cancer histology (http://kmplot.com/analysis/index.php?p=service&cancer=ovar).

### Cell lines and cell culture

A2780 cells were cultured in RPMI 1640 medium plus 10% fetal bovine serum (FBS). SKOV3 cells were cultured in McCoy’s 5 A plus 10% FBS. HEY and HEK293T cells were cultured in DMEM plus 10% FBS. All cell lines were confirmed using unique short tandem repeat (STR) analyses.

### RNA isolation and qRT–PCR

TRIzol reagent (Invitrogen, 15596018) was used to extract total RNA. PrimeScript RT master mix kits (Takara, RR037A) were used to obtain reverse RNA, and then the RNA was transcribed to generate cDNA. SYBR Green qPCR master mix (Takara, RR420A) was used to perform qRT–PCR. GAPDH was used as endogenous control. The primer sequences are listed in Table S1.

### Western blotting

RIPA lysis buffer (Beyotime Bio, P0013) was used to obtain protein lysates. The BCA Protein Assay Kit (Merck Millipore, 71287) was used for protein concentration determination. SDS–PAGE was used to separate protein samples, and the protein samples were transferred to PVDF membranes. Next, the membrane was incubated with primary antibody at 4 °C overnight. Then, the protein bands were incubated with secondary antibodies and detected with an ECL system (GE Healthcare). The antibodies used were as follows: SF3B4 (1:8000, Proteintech, 10482–1-AP), RAD52 (1:1000, Proteintech, 28045–1-AP), and β-actin (1:8000, Sigma–Aldrich, SAB3500350).

### RNA interference and lentiviral infection

miRNA mimics and small interfering RNAs (siRNAs) were purchased from GenePharma (Shanghai, China). ORFs of SF3B4 and RAD52 were purchased from Vigenebio (CH804949 and CH861367). shRNAs of SF3B4 were constructed based pLKO.1-TRC vector. HEK293T cells were used to package lentiviral vectors with psPAX2 (Addgene, 12260) and pMD2. G (Addgene, 12259). We infected OC cells with lentivirus for 24 h, and puromycin (2 µg/ml) was used for selection for 5–7 days. The sequences are shown in Table S2.

### Cell proliferation assay

To draw a growth curve, cells (1 × 10^3^) were seeded into 96-well plates. At the indicated times after seeding, the cells were incubated with 20 µl of MTT solution for 4 h at 37 °C. The absorbance at 450 nm was measured using a microplate spectrophotometer.

For the EdU cell proliferation assay (EdU), an EdU Kit (Beyotime, C0071s) was used. In brief, cells (1.5 × 10^4^) in 96-well plates were cultured with 1:1000 diluted EdU for at least 2 h. After that, the cells were fixed with 4% paraformaldehyde and stained with fluorescent dye and Hoechst.

### Colony formation assay

Cells (1 × 10^3^) were seeded into 6-well plates and then cultured for 10 days with medium. We used methanol to fix cells and 0.6% crystal violet to stain cells. Finally, we counted cell clones using Photoshop software.

### Immunohistochemistry (IHC)

For the immunohistochemical study, paraffin-embedded tissues were used to cut 4 μm thick sections. Tissue slides were deparaffinized and rehydrated. Antigen retrieval was performed by heating in a microwave in citrate buffer (pH = 6.0) or EDTA (pH = 9.0). The slides were incubated with 3% hydrogen peroxide for 20 min and then blocked with goat serum for 30 min. Next, the slides were incubated with primary antibody at 4 °C overnight. After that, the sections were incubated with the secondary antibody. Finally, the sections were stained with a DAB detection system. The final degree of immunostaining was evaluated based on the extent and intensity of staining. The antibodies used were as follows: SF3B4 (1:200, Proteintech, 10482–1-AP) and Ki-67 (1:600, CST, 9449 T).

### RNA-seq and bioinformatic analysis

We extracted total RNA from HEY cells after interference with SF3B4 siRNA and siNC using TRIzol reagent. Three replications of each sample were prepared for next-generation sequencing (Annoroad Genomics Co., Ltd, China). The raw data was uploaded to GEO database (GSE190310). The threshold for differential expression was |FoldChange | ≥1 and *p* < 0.05.

### Tumor xenograft models

Five-week-old female BALB/c nude mice in our research were purchased from Gem Pharmatech (Nanjing, China). Six mice in each group were injected subcutaneously with SF3B4-knockdown or control cells bilaterally. The mice were sacrificed, and the tumors were isolated, and the tumor weight and volume were measured. The expression of SF3B4 and Ki-67 in xenograft tumors was determined by IHC. The animal experiment conformed to Guide for the Care and Use of Laboratory Animals, and was approved by the Animal Care and use Committee of Shandong University (approval No. SDULCLL2019-2-08)

#### RNA immunoprecipitation

To analyze RNA molecules binding with SF3B4, an RIP Kit (Guangzhou Geneseed Biotech, P0101) was used to perform an RNA immunoprecipitation assay (RIP). Both input and RIP samples were prepared for qRT–PCR analysis as described in this section.

### Luciferase assay

The wild-type (WT) and mutant-type (MT) sequences of the SF3B4–3’UTR were cloned into primGLO plasmids. Then, HEK293T cells were transfected with SF3B4–3’UTR MT or WT vector and miRNA-509–3p mimics. A dual-luciferase reporter assay system (Promega, E2920) was used to measure luciferase activity 48 h after transfection.

### Statistical analysis

The Kaplan–Meier method was used to plot survival curves, and the log-rank test was used for survival analysis. Statistical significance was determined by Student’s t-test. All data were presented as the mean ± SD. SPSS 25.0 software was used to perform the statistical analysis. Results represent the mean ± SD of three independent experiments. *p* < 0.05 was considered significant.

## Supplementary information

Supplementary figure and tables

Author contribution

checklist

## Data Availability

The datasets used and/or analyzed during the current study are available from the corresponding author on reasonable request.
