# Supplementary material for: SF3B4 promotes ovarian cancer progression by regulating alternative splicing of RAD52
Source: Cell Death Dis. 2022 Feb 24;13(2):179. doi: 10.1038/s41419-022-04630-1 (PMC8873359; doi:10.1038/s41419-022-04630-1)
Supplement: Supplementary file 1 — Supplementary figure and tables [file 41419_2022_4630_MOESM1_ESM.docx]

**Supplementary figure and figure legends**

**Supplementary Figure. 1.** SF3B4 promotes proliferation, migration and invasion of A2780 cells. (A) qRT-PCR analysis of SF3B4 mRNA expression after SF3B4 knockdown (n=3 biologically independent samples). (B) Western blotting showed the SF3B4 protein levels after SF3B4 knockdown. (C, D) Growth curve and EdU assay evaluated the proliferation ability of A2780 cells upon SF3B4 knockdown (n=3 biologically independent samples). (E) The effect of SF3B4 downregulation on migration and invasion potential in A2780 cells (n=3 biologically independent samples). P value was obtained by Unpaired t-test. *P < 0.05, **P < 0.01.

**Supplementary Figure. 2.** Knockdown of RAD52 partially impair the proliferation and mobility of SF3B4 overexpressed ovarian cancer cells. (A) Transwell assay showed the migration ability of ovarian cancer cells upon RAD52 knockdown (n=3 biologically independent samples). (B) Different proliferation and migration capacity associated with different expression levels of SF3B4 and RAD52 in SKOV3 cells (n=3 biologically independent samples). P value was obtained by Unpaired t-test. *P < 0.05, **P < 0.01.

**Supplementary Figure. 3.** miR-509–3p suppress proliferation and invasion of ovarian cancer cells. (A) Schematic diagram showing miR-509–3p binding site in SF3B4 3’UTR region. (B) EdU assay and (C) clonogenic assay showed the proliferation capacity after miR-509–3p overexpression in ovarian cancer cells (n=3 biologically independent samples). (D, E) Transwell assay showed the migration and invasion ability of ovarian cancer cells upon miR-509–3p overexpression (n=3 biologically independent samples). (F, G) Different proliferation and migration abilities associated with different expression levels of miR-509–3p and SF3B4 in SKOV3 cells (n=3 biologically independent samples). P value was obtained by Unpaired t-test. *P < 0.05, **P < 0.01.

**Supplementary Figure. 4.** Representative IHC staining of SF3B4 in ovarian cancer.


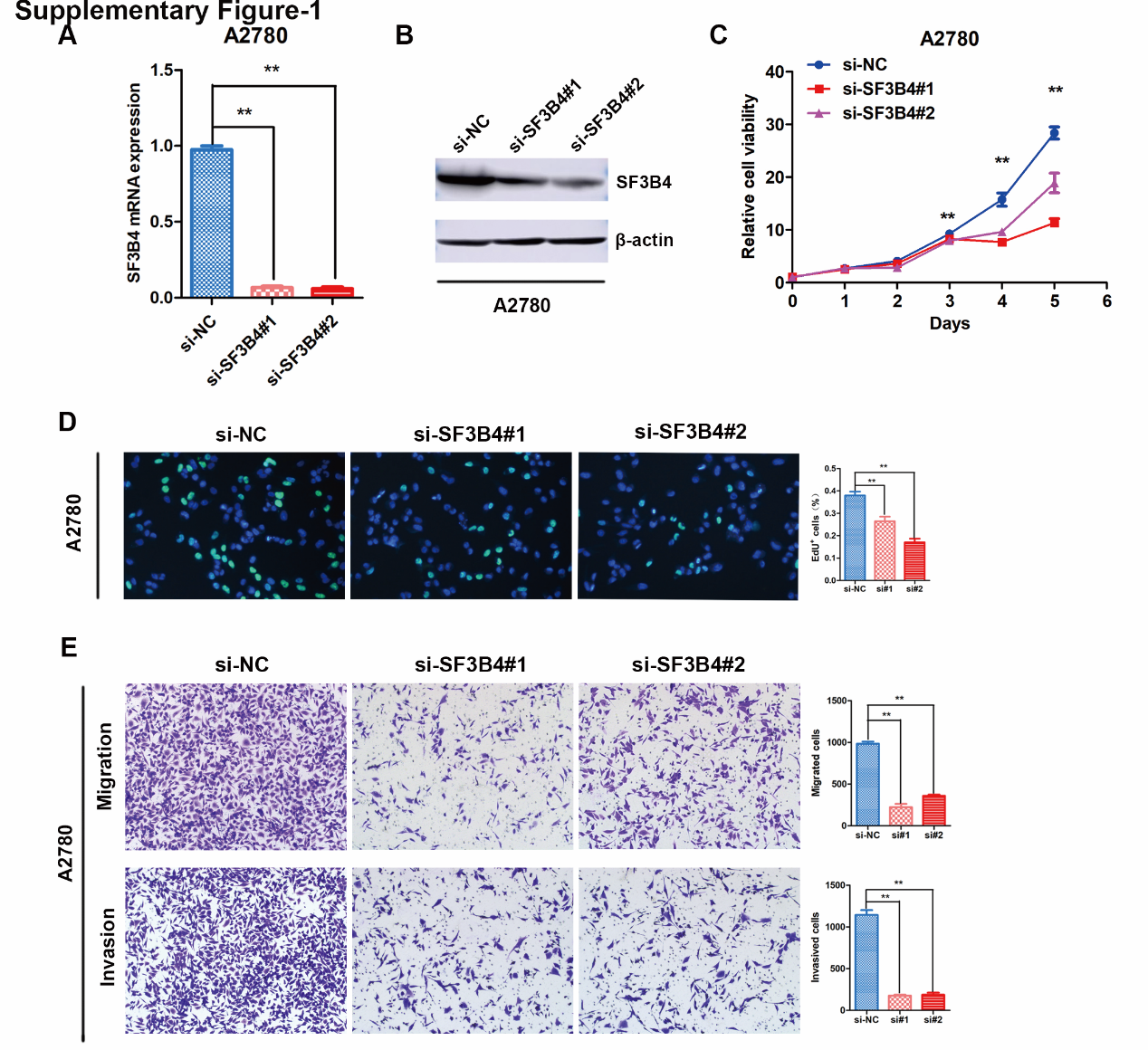


**
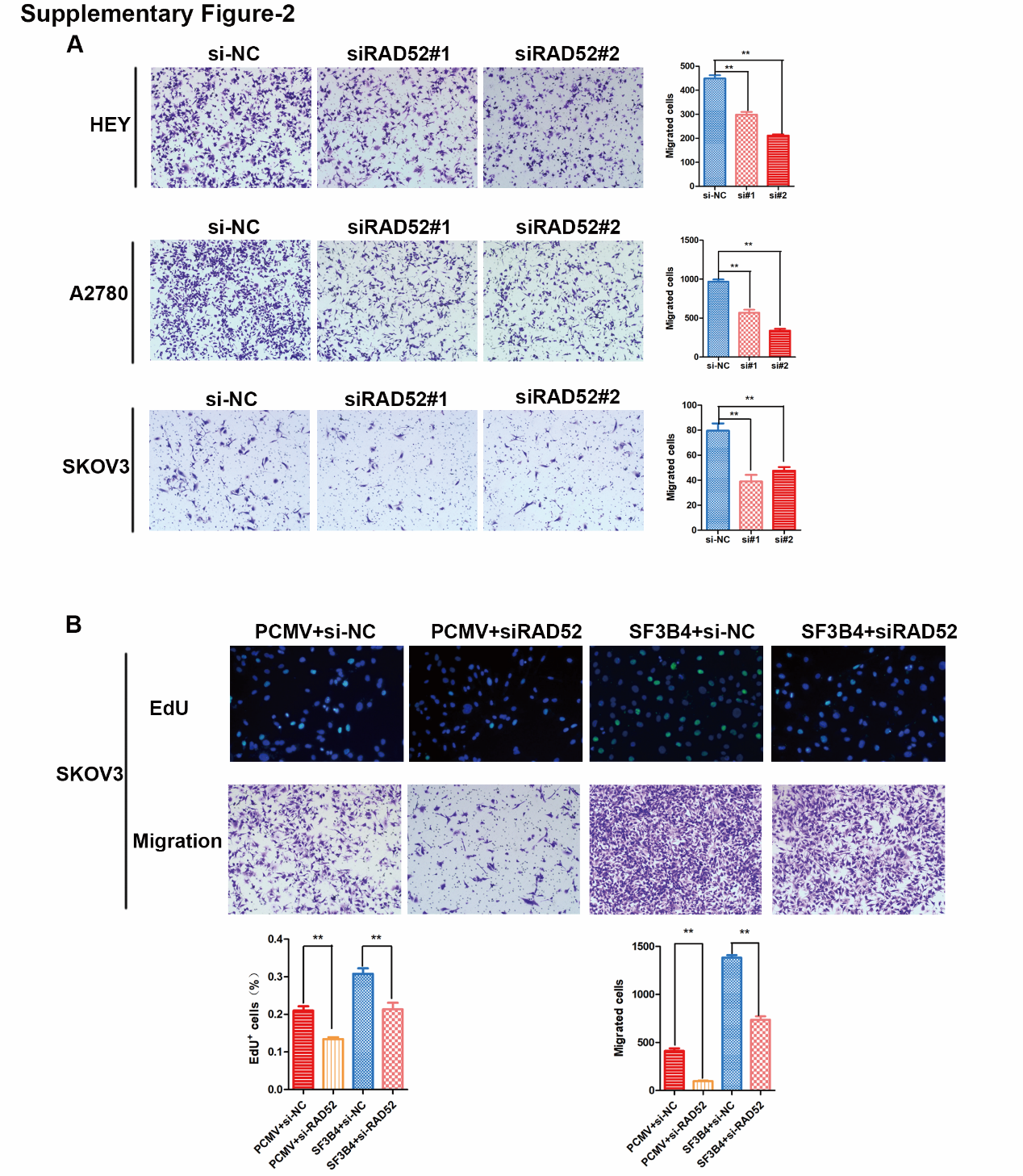
**

**
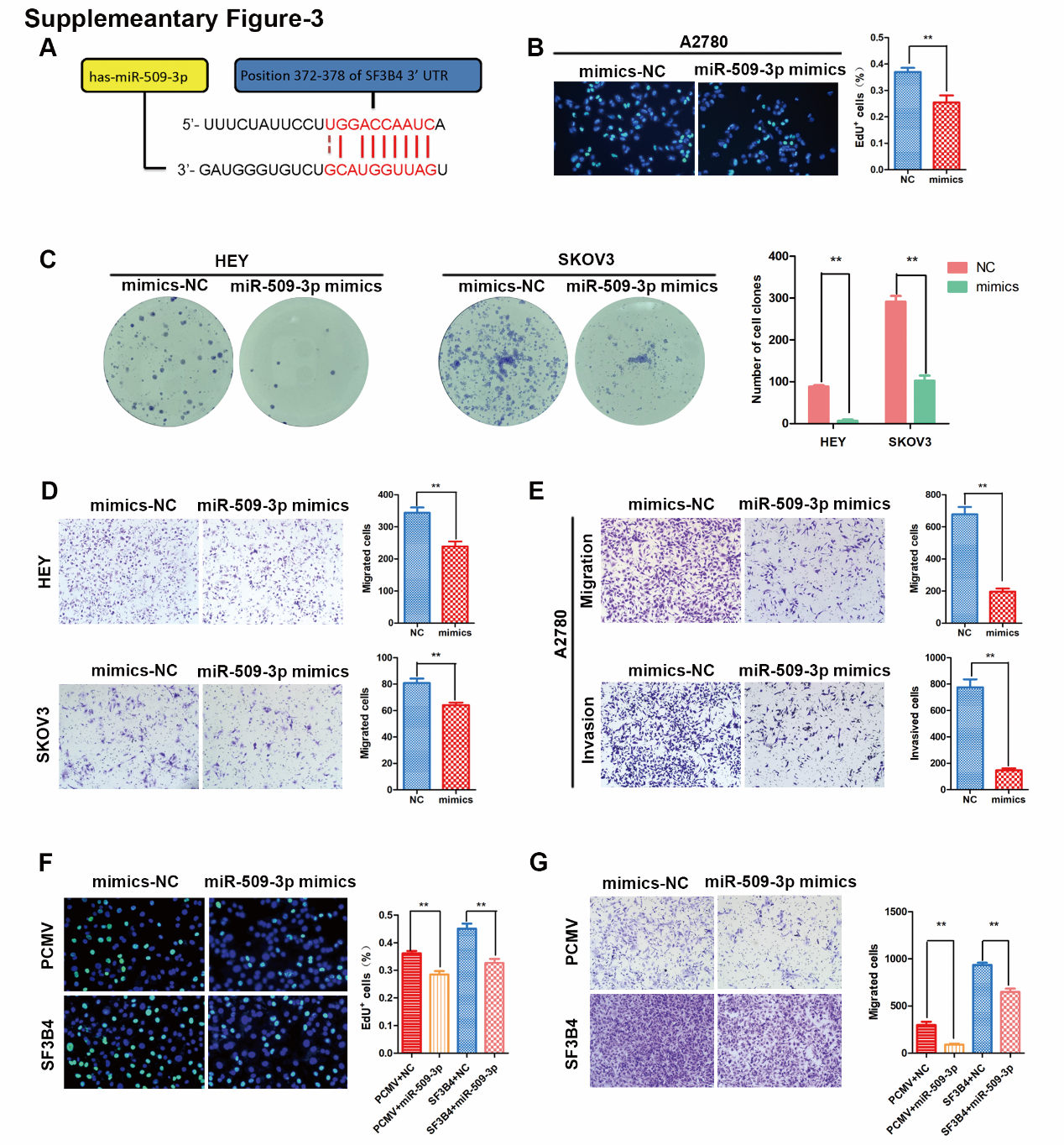
**

**
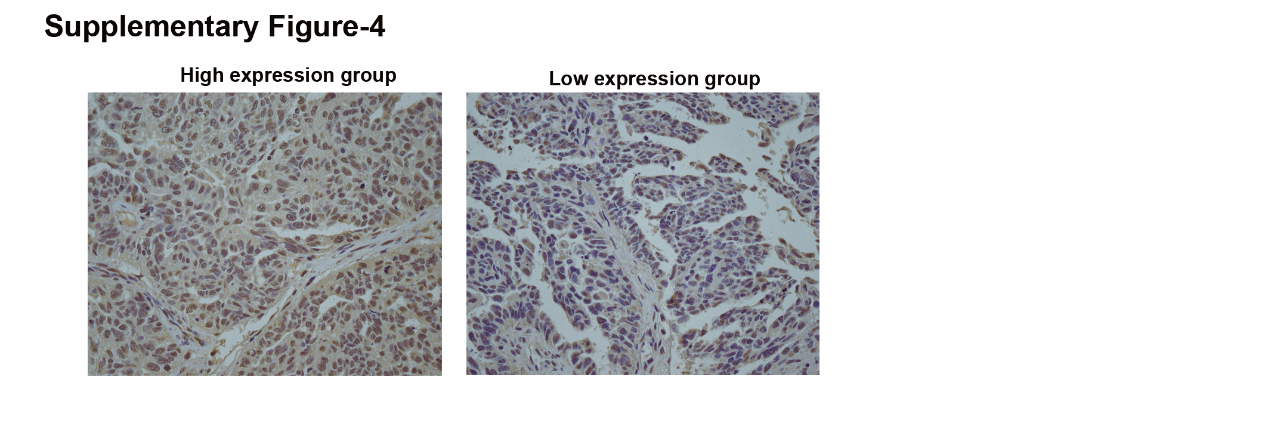
**

**Supplementary tables**

**Supplementary table 1.** Primer sequence used in this study.

**Supplementary table 2.** si-RNA, mimics and shRNA sequences used in this study.

**Supplementary table 3.** Wild type and mutant 3’-UTR sequences of SF3B4 used in this study.

**Supplementary table 1.** **Primer sequence used in this study.**

| Method | Name | Sequence (5’-3’) |
| --- | --- | --- |
| qPCR | GAPDH-F | GGTCTCCTCTGACTTCAACA |
| qPCR | GAPDH-R | GTGAGGGTCTCTCTCTTCCT |
| qPCR | SF3B4-F | AGTCAACACCCACATGCCAA |
| qPCR | SF3B4-R | CACCCGTATTGGCTTCCCAT |
| qPCR | RAD52-F | GAGCCCTCAGGAGTTTTGG |
| qPCR | RAD52-R | TCCACTTCAAGAGGCAACTG |
| qPCR | RREB1-F | ACCAAATCGGATTGGCAGAAG |
| qPCR | RREB1-R | GGTGCTGGGTAGTGCAAATCT |
| qPCR | STAT2-F | CAGGTCACAGAGTTGCTACAGC |
| qPCR | STAT2-R | CGGTGAACTTGCTGCCAGTCTT |
| qPCR | DDX11-F | CCTTTGGCAAGGATGTTCGGCT |
| qPCR | DDX11-R | TGTCCACACAGCGGTCGTTGAT |
| qPCR | ABL2-F | TGAAAAGCTACGAGTCCTTGGT |
| qPCR | ABL2-R | GCTGTTCACTGGGGTGATGTA |
| qPCR | FNBP1L-F | CTTGCCTGCTATTGGACACTGC |
| qPCR | FNBP1L-R | GCTCTTGTCCATCCGTCACCTT |
| qPCR  qPCR  qPCR  qPCR  qPCR  qPCR  qPCR  qPCR  qPCR  qPCR  qPCR  qPCR  qPCR  qPCR  qPCR  qPCR  qPCR  qPCR  qPCR  qPCR  qPCR  qPCR  qPCR  qPCR  qPCR  qPCR  qPCR  qPCR  qPCR  qPCR  qPCR  qPCR  qPCR  qPCR  qPCR  qPCR  qPCR  qPCR | ADAM17-F  ADAM17-R  NUP62-F  NUP62-R  BBS1-F  BBS1-R  SLC39A9-F  SLC39A9-R  AAMP-F  AAMP-R  PITPNM1-F  PITPNM1-R  DZIP3-F  DZIP3-R  DDX51-F  DDX51-R  CAPRIN2-F  CAPRIN2-R  ZGPAT-F  ZGPAT-R  TPP1-F  TPP1-R  HFE-F  HFE-R  HIP1R-F  HIP1R-R  ZMYM2-F  ZMYM2-R  CEP170-F  CEP170-R  ABCC5-F  ABCC5-R  ATG3-F  ATG3-R  RBM25-F  RBM25-R  PKD1-F  PKD1-R | TTTCACGTTTGCAGTCTCCAA  AGAAGCGATGATCTGCTACCA  GGAACAGCGACTCTTGCTTC  GGTGCTCGATATGGCATTAGTG  CCTGCTCAATGTCATCCACACC  CACTGCTGTACGCTTCAGGATC  GCAGCATCTACTTCACAGACCAG  GATTCCGCTCTAAGCCAGCATG  GGAAGAGAGCTGTGGTAGGCTA  GTTGGCAGCAACACAGGTGAGT  TCTACTAGCGGTGCCTTCTGGA  CAGTACAGCGAGTAGTCGATCC  GGGACCTCAATACCCAGTGAATC  CTCAACTGCTCTTCCTTTTCTACC  AGATGCCACACCTCTGAGAGTC  CTACCACGATGTCAGCCAAGCA  CACAGATTTCTCCAAAGTCCTGG  GAGGAAGAACCTACAGGCTGATG  CTCCTCACCAAGATGGGCTATG  TTGCCAACCCTGGTCTGCTTCT  GGTGGCTTCAGCAATGTGTTCC  GAAGTAACTGGATGGTGGCAGG  GTCAGAGTCTGAAAGGGTGGGA  TTTCACAGCCCAGGATGACCTG  AGATGCTGTGCGGAGGATTGAG  TGCAGGCTAGTGGATGTCGTCA  TGTTCCAGTGCCTATCCCTGTG  TCTCACTGCTGTCCAATGGAGC  GCTACAGAAGGTCCAGGCTACT  TGACACCGATGGCTCCTCTGAT  GGCTGTATTACGGAAAGAGGCAC  TCTTCTGTGAACCACTGGTTTCC  ACTGATGCTGGCGGTGAAGATG  GTGCTCAACTGTTAAAGGCTGCC  GTTTCCTCTGCCAGTGGCAATG  CCTATGCTCCTCAGGTTGCTGT  AGCAGCACGGTCACCATTCCAC  CACTCCAAGGACACAATGGGCA |
| RT-PCR | RAD52-F | AGATCTTGAACCGTCTGTGGA |
| RT-PCR | RAD52-R | TCACTCTTCTCAGCTGACGG |

**Supplementary table 2. si-RNA, mimics and shRNA sequences used in this study.**

| Method | Name | Sequence (5’-3’) |
| --- | --- | --- |
| si-RNA | si-SF3B4#1 | GGAUGAGAAGGUUAGUGAATT |
| si-RNA | si-SF3B4#2 | GCACCAAGGCUAUGGCUUUTT |
| si-RNA | si-RAD52#1 | UGAAGUGGAUUUAACUAAATT |
| si-RNA | si-RAD52#2 | CUUGGGACCUCCAAACUUATT |
| si-RNA | negative control | UUCUCCGAACGUGUCACGUTT |
| mimics | miR-509-3p mimics | UGAUUGGUACGUCUGUGGGUAG |
| sh-RNA | sh-SF3B4-F1 | CCGGCCCTGAGATTGATGAGAAGTTCTCGAGAACTTCTCATCAATCTCAGGGTTTTTG |
| sh-RNA | sh-SF3B4-R1 | AATTCAAAAACCCTGAGATTGATGAGAAGTTCTCGAGAACTTCTCATCAATCTCAGGG |
| sh-RNA | sh-SF3B4-F2 | CCGGGGATGAGAAGGTTAGTGAACCCTCGAGGGTTCACTAACCTTCTCATCCTTTTTG |
| sh-RNA | sh-SF3B4-R2 | AATTCAAAAAGGATGAGAAGGTTAGTGAACCCTCGAGGGTTCACTAACCTTCTCATCC |

**Supplementary table 3.** **Wild and mutant 3’-UTR sequences of SF3B4 used in this study.**

| Name | Sequence |
| --- | --- |
| SF3B4-3’UTR-WT | AGGCCCTCTCCCTCAGTAAATTCACATTTTCCTTCCTCCTGTTACATTTTCCCAATATCTTTTCTATTCCTTGGACCAATCAGAGATGCTGTAGCTCCTTGGGGCAAAGGTACTAATCCCTTTCAGCACCCCCACTCCATTCCCCTTTTTAATGTAACTTTTTCCACAGGAGGTATTTCTTTTTTATGTTGGTCCTGAGTATTTTGCAAATGCACAGAGAAAATA |
| SF3B4-3’UTR-MT | AGGCCCTCTCCCTCAGTAAATTCACATTTTCCTTCCTCCTGTTACATTTTCCCAATATCTTTTCTATTCCTTGGAGAGATGCTGTAGCTCCTTGGGGCAAAGGTACTAATCCCTTTCAGCACCCCCACTCCATTCCCCTTTTTAATGTAACTTTTTCCACAGGAGGTATTTCTTTTTTATGTTGGTCCTGAGTATTTTGCAAATGCACAGAGAAAATA |
