# Supplementary material for: SF3B4 promotes ovarian cancer progression by regulating alternative splicing of RAD52
Source: Cell Death Dis. 2022 Feb 24;13(2):179. doi: 10.1038/s41419-022-04630-1 (PMC8873359; doi:10.1038/s41419-022-04630-1)
Supplement: Supplementary file 2 — Author contribution [file 41419_2022_4630_MOESM2_ESM.pdf]

DECLARATION OF CONTRIBUTIONS TO ARTICLE

ADMC

Manuscript Number:	Journal Name:	
CDDIS-21-4026	*Cell Death & Disease*	(the 'Journal')

Proposed Title of the Contribution:

SF3B4 promotes ovarian cancer progression by regulating alternative splicing of oncogenic RAD52 (the 'Contribution)

Author(s):

Yuchao Diao, Zixiang Wang，Shourong Wang, Peng Li, Yingwei Li, Beihua Kong (the 'Authors')

For all *CDDis* articles, each person named as an author in the published version must be able to show he or she has contributed substantially to the article.

Authorship credit should be based on 1) substantial contributions to conception and design, acquisition of data, or analysis and interpretation of data; 2) drafting the article or revising it critically for important intellectual content; and 3) final approval of the version to be published. Authors should meet conditions 1, 2 and 3.

Any person who cannot be shown to have made a substantial contribution to the article cannot be listed as an author in the final version. The name of any person who is deemed to have made a minor contribution can, however, appear in the Acknowledgments section of the article.

Please complete the table below to indicate the contributions of all named authors to the manuscript.

Author Full Name:	Specification of Contribution to the Manuscript:
Yuchao Diao	Methodology; Acquisition of data; Analysis and interpretation of data;Administrative, technical, or material support;Writing, review, and/or revision of the manuscript; Final approval of the manuscript
Zixiang Wang	Methodology; Acquisition of data; Analysis and interpretation of data;Administrative, technical, or material support;Final approval of the manuscript
Shourong Wang	Methodology; Analysis and interpretation of data;Administrative, technical, or material support; Final approval of the manuscript
Peng Li	Administrative, technical, or material support; Final approval of the manuscript
Yingwei Li	Conception and design; Study supervision;Methodology: Analysis and interpretation of data;Administrative, technical, or material support;Writing, review, and/or revision of the manuscript; Final approval of the manuscript
Beihua Kong	Conception and design; Study supervision; Final approval of the manuscript

Please complete the table below to indicate the contributions of all named authors to the figures.

Figure 1:
B.K ,Y. L and Y.D generated the data and prepared the Figure; Z.W and S.W labelled the image.

Figure 2:
Y.D generated the data; P.L labelled the image. Y. L assembled the figure.

Figure 3:
Y.D generated the data; P.L labelled the image. Y. L assembled the figure.

Figure 4:
Y.D generated the data; P.L labelled the image. Y. L assembled the figure.

Figure 5:
Y.D generated the data; Z.W labelled the image. Y. L assembled the figure.

Figure 6:
Y.D generated the data; S.W labelled the image. Y. L assembled the figure.

Signed for and on behalf of the Author(s): Yuchao Diao Zixiang Wang Shourong Wang Peng Li Yuchao Diao Yingwei Li Beihua Kong

Print Name: Yuchao Diao, Zixiang Wang, Shourong Wang, Peng LI, Yingwel LI, Beihua Kong

Date: 2021/10/07
